# Oral health and healthy ageing: a scoping review

**DOI:** 10.1186/s12877-023-04613-7

**Published:** 2024-01-08

**Authors:** Prakash Poudel, Grish Paudel, Reecha Acharya, Ajesh George, Wenche S. Borgnakke, Lal B. Rawal

**Affiliations:** 1 Office of Research and Education, Canberra Health Services, Australian Capital Territory (ACT) Government, Garran, ACT 2606 Australia; 2 Australian Centre for Integration of Oral Health (ACIOH), School of Nursing & Midwifery, Western Sydney University, Liverpool, Australia; 3 Ingham Institute for Applied Medical Research, Liverpool, Australia; 4 School of Health, Medical and Applied Sciences, Central Queensland University, Sydney, NSW 2000 Australia; 5 Appleton Institute, Physical Activity Research Group, Central Queensland University, QLD, Rockhampton, 4702 Australia; 6 School of Dentistry, Faculty of Medicine and Health, The University of Sydney, Camperdown, NSW 2006 Australia; 7 School of Nursing, Faculty of Science, Medicine & Health, University of Wollongong, Wollongong, NSW 2522 Australia; 8 Department of Periodontics and Oral Medicine, University of Michigan School of Dentistry, Ann Arbor, MI USA; 9 Department of Periodontics and Preventive Dentistry, University of Pittsburgh School of Dental Medicine, Pittsburgh, PA USA; 10 Translational Health Research Institute (THRI), Western Sydney University, Sydney, NSW 2751 Australia

**Keywords:** Aged, Health policy, Dental care for aged, Patient care team, Integrated health care systems, Mastication, Health status

## Abstract

**Background:**

Good oral health is an important part of healthy ageing, yet there is limited understanding regarding the status of oral health care for older people globally. This study reviewed evidence (policies, programs, and interventions) regarding oral health care for older people.

**Methods:**

A systematic search of six databases for published and grey literature in the English language by the end of April 2022 was undertaken utilising Arksey and O’Malley’s scoping review framework.

**Results:**

The findings from oral health policy documents (*n* = 17) indicated a lack of priorities in national health policies regarding oral health care for older people. The most common oral health interventions reported in the published studies (*n* = 62) included educational sessions and practical demonstrations on oral care for older adults, nurses, and care providers. Other interventions included exercises of facial muscles and the tongue, massage of salivary glands, and application of chemical agents, such as topical fluoride.

**Conclusion:**

There is currently a gap in information and research around effective oral health care treatments and programs in geriatric dental care. Efforts must be invested in developing guidelines to assist both dental and medical healthcare professionals in integrating good oral health as part of healthy ageing. Further research is warranted in assessing the effectiveness of interventions in improving the oral health status of the elderly and informing approaches to assist the integration of oral health into geriatric care.

## Background

The ageing population worldwide is increasing rapidly. Worldwide, there were over 703 million (9%) people aged 60 years or older in 2020, and expected to increase to 1.5 billion (16.0%) by 2050 [1]. The population is ageing in low-and middle-income countries (LMICs) as well as in high-income countries (HICs) [1, 2], however, the majority (> 80%) will be living in LMICs by 2050 [2]. Ageing is often associated with physiological changes, low appetite, and nutritional problems. Chronic diseases and any medications taken for them may result in a decrease in salivary flow (hyposalivation), leading to chewing (mastication) and swallowing difficulties (dysphagia), which may prevent adequate nutritional intake [3–5]. People with dysphagia are also reported to have a lower quality of life. Further, hyposalivation can lead to a greater incidence of coronal and root caries and periodontitis, which ultimately may lead to tooth loss [6, 7] that in turn negatively impacts chewing function, aesthetics, self-perception, and quality of life. Periodontitis, the prevalence of which also increases with age, affects the majority of seniors [8, 9], is causally linked with aspiration pneumonia, resulting in morbidity, hospitalization, and mortality [8]. Further, poor oral health is linked to, and compounded by, the development of several systemic health conditions, such as cardiovascular disease [3, 10] and diabetes mellitus [11–13], and all which compromise the health of older people.

Globally, the share of chronic and non-communicable diseases (NCDs), such as cardiovascular diseases, cancer, diabetes, and chronic obstructive pulmonary diseases (COPD), is substantially greater among older populations than in younger age groups [14, 15]. Oral diseases share the common risk factors such as unhealthy diet high in sugar, use of tobacco, and harmful use of alcohol with these other major NCDs [3, 10]. Older populations with NCDs are also more likely to develop oral health problems and those with poor oral health conditions are likely to manage their NCDs poorly. Ageing population are likely to develop NCDs which have a negative impact on oral health [16], that is associated with overall health, comfort, dignity, and well-being [17].

Poor oral health, including dental caries, periodontal diseases and the resulting tooth loss, induce a change in food selection and dietary patterns, potentially resulting in frailty and dependency [18–20], subsequently affecting general well-being and quality of life [21]. There are several factors causing poor oral health outcomes among the older population. A recently conducted systematic review identified some key determinants to oral health frailty among older populations, including oral health status deterioration, few remaining teeth, reduced oral motor skills, oral diadochokinetic and chewing, swallowing, and saliva disorders [22].

Improving oral health outcomes of the older population requires adequate oral health interventions tailored to the specific needs of this age group that will influence their quality of life [23, 24]. Improvement in oral health status can have a significant positive impact. For example, older people with more natural, intact teeth have reported a perceived greater quality of life, positive body image, increased self-worth, maintenance of oral function, and sense of achievement [25, 26]. However, there are currently several barriers to receiving oral healthcare for the older population, such as limited access to professional oral health care providers. Furthermore, geriatric dentistry is not a recognized specialty in most countries [27]. Within this context, innovative oral health promotion programs that co-manage oral health problems with other healthcare providers may be important to address the unmet needs of older populations. Oral health promotion programs at a primary care level can play a significant role in maintaining better overall health and quality of life outcomes for the aging population. However, there is limited understanding in these areas. Moreover, there is also a paucity of information on programs that have involved non-dental professionals to address the oral health problems of older populations [28].

Some programs do exist, for instance one in Australia, where Registered Nurses (RNs) and General Practitioners (GPs) involved in aged care settings across the country were trained to promote oral health, with this program successfully being rolled out to more than 80% of residential aged care homes nationally. However, a thorough synthesis of evidence available regarding oral health care for older people has yet to be undertaken. Furthermore, it appears that there is limited among older populations knowledge and understanding of oral health care for older people residing in LMICs [29].

### Objectives

This scoping review was undertaken to identify and synthesise the existing evidence on the following aspects:

- oral health policies, strategies and guidelines discussing about promoting oral health in older people, and
- interventions/programs that have been implemented to improve oral health of older people.

## Methods

This review was conducted using a scoping review framework first outlined by Arksey and O’Malley [30] and later revised [31, 32]; and findings are reported in accordance with the Preferred Reporting Items for Systematic reviews and Meta-Analyses extension for scoping reviews (PRISMA-ScR) checklist [33]. The application of a scoping review format aids in identifying available peer-reviewed and grey literature in this area and to identify gaps in the pertinent evidence base [30]. The scoping design further allows for an iterative process that will facilitate a more comprehensive review of the literature compared to applying a priori fixed criteria [30]. As scoping reviews aim to map existing evidence on the topic and not produce critically appraised and synthesised results, it is not essential to undertake quality assessment of included studies [30]. Finally, a scoping review provides a basis for assessment of the relevance and feasibility of subsequently conducting a systematic review, in this case regarding assessment and synthesis of the best evidence to promote oral health of older people in primary health care settings.

### Eligibility criteria

Published or unpublished/grey literature available as full text in the English language by the end of April 2022 that addressed at least one of the oral health areas: policies, guidelines, or programs, and interventions for improving oral health of the older people, were eligible for inclusion, with no restrictions regarding study design, settings, or quality.

### Information sources

Individually adapted systematic search strategies were designed and conducted among records of published literature in the following six databases: PubMed, CINAHL, EMBASE, Cochrane Database of Systematic Reviews, Scopus, and ProQuest. Individual search strategies were developed for each database in consultation with an experienced information specialist (a. k. a. librarian). Grey literature was searched using Google Scholar and ProQuest Dissertation and Thesis and also through webpages of relevant organisations and agencies, such as the WHO NCD Documents Repository (https://extranet.who.int/ncdccs/documents/Db) and International Association of Gerontology & Geriatrics (IAGG) (https://www.iagg.net/). In addition, the reference lists in relevant papers were reviewed manually to identify additional publications on the topic.

### Search strategy

The search strategies included use of combination of keywords/free text terms and Medical Subject Headings (MeSH) using Boolean operators. Use of diverse terminology and the spelling of keywords were considered to aid in the identification of relevant literature using truncations and wildcards. As per the focus of the scoping questions, the following keywords were used in the search under the following PICO (population, intervention, context/comparison/control, and outcomes) terms [34].

#### Population

Aging, OR aging population OR aged OR elder OR older OR geriatric OR aged care OR residential care OR nursing home OR care home.

#### Intervention

Policies OR strategies OR guidelines OR practice guidelines OR intervention OR trial OR health promotion OR health program OR preventive services OR management services OR recommendation OR consensus OR resolution.

#### Context/comparison/control

*N/A.*

#### Outcomes

Oral hygiene OR oral health OR tooth brushing OR interdental cleaning OR flossing OR dental visit OR dental check-up OR nutritional intake OR nutritional status OR malnutrition OR nutritional assessment OR quality of life OR QoL OR oral health related quality of life OR OHRQL OR mastication OR chewing ability OR oral function OR swallowing ability OR diadochokinesis OR oral frailty OR cognitive status OR frailty.

### Selection process

After removal of duplicates, the titles and abstracts were screened for fulfillment of the inclusion/exclusion criteria. The full texts of potentially eligible scientific reports were retrieved and independently reviewed by two authors (GP, RA) for inclusion. Further, national policies, guidelines, and strategies from any country or geographic region focusing on oral health aspects of older persons were reviewed for their relevance to the aim of the study. Any discrepancies between reviewers during the screening process was resolved through discussion and consensus with other authors (PP, LBR). Similarly, the full texts of the articles were reviewed (GP, RA), and were discussed with the other authors (PP, LBR).

All literature and policy documents relating to at least one of the focus areas were included in the review.

### Data collection process

Data on key information relating to the stated aims were extracted, including author name, publication year, country, study focus/aim, study design, intervention, study setting, sample characteristics and size, key findings, conclusions, and recommendations. Data were collated, summarised, and narratively reported, using synthesizing text and presented in table form by two authors (RA & GP) under the guidance of (PP and LBR). The methodologic quality of the studies were not assessed, as the focus of this scoping review was to synthesise and present the current evidence on this emerging topic, regardless of study design and quality, which would be assessed in a future systematic review, if warranted.

### Data items (outcomes)

The data items/outcomes relating to policy included: access to oral health care services and education (training) in oral health care of older population. In the published studies, the outcomes included interventions implemented in improving oral health problems or oral health status at a) nursing homes/RACFs/long-term care settings b) hospitals/clinics/other health facilities, and c) community-based settings.

## Results

The results of this scoping review are presented in the following two focus areas: (A) Evidence from policy documents and guidelines/strategies regarding oral health for the older population; and (B) Evidence from the scientific literature concerning preventive and therapeutic interventions relevant to the oral health of the older population.

### A) Selection of policy documents and guidelines/strategies regarding oral health for the older population

A total of 104 records of policy documents from 71 countries were retrieved through the WHO data repository [35]. Of these, 66 were excluded due to publication language other than English (*n* = 60) or unavailability of the full text (n = 6). Of the remaining 38 documents, 17 specified oral health aspects of the older population [36–52]. The majority (*n* = 9) of the policies were from HICs, including Canada (*n* = 2), Ireland (*n* = 1), Australia (n = 1), Japan (n = 1), Barbados (n = 1), Cook Islands (n = 1), New Zealand (n = 1), and Trinidad and Tobago (n = 1) [36, 37, 40, 41, 43, 46, 47, 50, 52].

The findings from the review of the 17 included policy documents are presented under the following two broad topics, namely *access to oral health care services* and *provision of oral health training* (Table 1).

**Table 1:** Policy documents on oral health and healthy ageing (*N* = 17)

Country/Organization: Year [Ref. #] Document Type	Aim/Purpose	Key Priorities/ Focus Areas	Strategies/Recommendations
Canada: 2005 [40], Strategy	Improve overall OH of Canadians by identifying inequalities, disparities and barriers to achieving optimal OH	• Foster public awareness of the importance of OH • Improve access to OH services	• Training and education for practitioners in OH care of elderly • Follow-up the cases of dental neglect among elderly to improve access as well as reduce barriers to OH care
New Zealand: 2006 [41], Policy	Develop OH policy for older adults	• Determine the needs of 4 older groups: independent older adults, moderately dependent older adults, highly dependent older adults, and older adults from groups experiencing inequalities (both independently and as part of the other three groups)	• Building the OH workforce to aid in providing OH services to older adults and category-specific (such as independent older adults, moderately dependent older adults, highly dependent oral adults and older adults experiencing particular inequalities) • OH needs and care to be explored
Uganda: 2007 [42], Policy/Guideline	Improve OH of Ugandans for general well-being and quality of life	• Integration of OH policy elements and strategies into programmes and policies of all sectors that have impact on community health including elderly	N/A
Barbados: 2009 [50], Policy	Improve OH of Barbadians by minimising the incidence of OH problems	• OH promotion and prevention, • Human resource development and OH information system	• Provision of OH education and intervention to all age groups • Prevention/early detection of oral cancers • Promote linkage of OH through planned communications
Trinidad and Tobago: 2010 [43], Policy	Provide framework for implementation of OH strategies for the formulation of quality, acceptable, equitable and cost-effective OH care services	• Decrease rate of dental decay, oral cancer mortality rate; • Decrease proportion of edentulous people over 65 years	• Elderly (65+ years) categorised as vulnerable group • Need to be provided a full range of OH care services-preventive, diagnostic, curative, as well as referral whenever required
Japan: 2011 [52], Act	principles to promote the maintenance of oral health and also to implement measures related to the promotion of Dental and Oral Health in a comprehensive manner	• Promote dental and oral health	• Encourage people to work towards prevention of dental diseases in daily life
Canada: 2013 [46], Strategy	Provide a framework to review the OH gaps and to prevent/control oral diseases	• Good access to OH care for long term care residents	• Focus on daily oral hygiene, periodontal disease, routine health assessments, and treatment of failing dental restorations, • Regular OH screening programs, preventive services as well as referrals for treatment in long term care facilities
Grenada: 2014 [51], Policy	Launch special OH care education programmes	• Educate elderly and their caregivers/families on preventing OH issues	• Special OH programme for older people (> 60) and caregivers /families of elderly person to educate them on preventing OH issues
Timor-Leste (East Timor): 2015 [39], Strategy	Promote OH and primary prevention as well as improve the quality, accessibility, effectiveness, and sustainability of the OH services	• Focus on fluoridation • improve access to OH services; strengthen and utilize the manpower to promote OH; deliver quality OH care	• Making fluoridated toothpaste accessible and affordable to everyone, including the elderly • Ensuring equality and accessibility to OH care
Malaysia: 2017 [44], Strategy	Address key areas of OH concerns to improve the overall OH of Malaysians	• Increase preventive OH behaviours among older populations	• Promote preventive dental visits among older populations • Promote good OH practices among older population at home like brushing and flossing
Nigeria: 2017 [45], Policy/Strategy	Achieve optimal OH for Nigerians through sustainable awareness creation, early detection, and prompt treatment of OH diseases	• Integration and promotion of OH as a part of general health Equitable, accessible, affordable, and appropriate OH for all	• Basic OH care services to be provided to priority populations that includes elderly • Understanding barriers to OH care of the target population and focusing on facilitators to meet their needs
Australia: 2017 [47], Strategy	Improve overall well-being of Australians by improving OH* and reducing the burden of OH diseases	Older adults (65+ years) in priority populations	• Older adults should receive OH check-up • Preventive OH care at least every 2 years • Integrate OH risk assessment in routine general health assessment
Jamaica: 2017 [49], Policy	Ensure that every elderly individual should receive quality OH care at an affordable cost.	• Education to seniors and care providers as well as policy advisors on importance of OH • Supporting long term care facilities with space for dental equipment to provide treatment on site	• Access to quality OH care at an affordable cost • Emphasis on correlation of OH with general health to improve the overall quality of life • Focus on preventive OH programmes for adults such as dental prophylaxis (cleaning), fluoride varnish, as well as oral cancer screening and OH education programmes.
Rwanda: 2019 [48], Strategy	Provide a strategic approach to prevent, treat, control, and reduce the burden of OH disease 2016–2024)	• Ensure universal (geographical and financial) access to quality OH services inclusive of preventive, curative, rehabilitative, and promotional OH care for all	• Introduce preventive OH measures to elders, who are categorised as priority population.
Cook Islands: 2019 [36], Strategy	Improve service delivery for elderly population	• Provide rehabilitative services to elderly. • Improve oral and general well-being of the elderly	• Improve rehabilitative health services to elderly population • Oral functions restored in people with compromised dentition
Ireland: 2019 [37], Policy	Ensure access to appropriate health care professionals to supplement primary OH care services for more vulnerable population, inclusive of the older population	• Access to OH services in primary health care setting	• Development of model of care specific to older population to access and navigate the health system in cost-effective setting
Vanuatu: 2019 [38], Policy	Improve access to quality, affordable, timely, person-centred, minimally invasive, and effective OH care services	• OH promotion, facility based OH services, equitable access, local effective OH strategies, information and research, collaboration between partners	N/A

*OH* Oral health, *N/A *Not appliccabe, *Ref. # *Reference number

#### Access to oral health care services

Irelands’ National Oral Health Policy discusses the need to ensure access to appropriate health care professionals to supplement primary oral health care services for more vulnerable populations, including older people [37]. The policy specifically depicts the development of models of care specific to the older populations ages 65–69 and > 70 years [37]. Australia’s National Oral Health Plan 2015–2024 [2015] emphasized that all older adults should be able to receive an oral health check-up and preventative oral health care at least every 2 years [47]. The plan also stipulates that the oral health risk assessment should be included as a component of the general health assessment. Furthermore, it highlights that an oral health care plan must be developed and implemented in conjunction with a dental practitioner for those entering aged care facilities [47].

New Zealand’s Strategic Vision for Oral Health [2006] aims to develop oral health policy for older adults [41]. This initiative is intended to aid in providing oral health services for older adults by focusing on building the oral health workforce, such as community based general dentists. Furthermore, it highlights a need to develop group specific strategies to promote the oral health of older adults, such as, to independent older adults, moderately dependent older adults, and highly dependent older adults [41]. Japan’s Oral Health Act [2011] emphasized ensuring preventive as well as t oral health services for the older population [52]. Jamaica’s Oral Health Policies & Business Plan for the Repositioning of Oral Health Services [2017] highlighted that older people should have access to high quality oral health care at an affordable cost [49]. It also highlighted the importance of maintaining good oral health, which correlates with general health, for improved quality of life. Furthermore, the policy emphasizes the importance of oral disease prevention programs, such as dental prophylaxis (cleaning) to prevent gum disease, as well as application of fluoride varnish and dental sealants to prevent caries. The Cook Island’s National Oral Health Strategy (2014–2018) focused on providing rehabilitative oral care services to older people, restoring their oral functions and improving the oral health as well as overall health [36].

Similarly, this review identified policies from different countries, namely, Canada [46], Trinidad and Tobago [43], Nigeria [45], Vanuatu [38], Malaysia [44], Timor-Leste [39], and Rwanda [48] that shared a common goal of improving access to quality, affordable, timely, preventative, and effective oral health services. The Uganda’s National Oral Health Policy (2007) lacked in providing information regarding the definitive aspects of older population’s oral health care [42].

#### Provision of oral health training

Oral health policy documents from some countries, namely Jamaica [49], Barbados [50], Grenada [51] and Canada [46] stated the need to foster public and/or health care professionals’ awareness regarding the importance of oral health care. These policies also highlighted the relationship of oral health with overall well-being among the older population [40, 49–51]. The Canadian Oral Health Strategy (2005) stated the needs for educating oral health practitioners regarding oral health care as well as focusing on follow-up of the cases of dental neglect and studying the barriers to oral health care of older populations can improve the target population’s accessibility to oral health services [40]. Furthermore, the policy mentioned that this can in turn facilitate the aim of this initiative to improve the overall oral health of Canadians by identifying inequalities in the system, disparities in health, and barriers to achieving optimal oral health [40]. Barbados’ Oral health policy (2009) stated the needs for improving the oral health of Barbadians by minimising the rate of oral health problems and unmet oral health needs through public education for all age groups, including the older population, and early detection and treatment/prevention of oral health diseases, such as oral cancer [50]. Likewise, Grenada’s Oral Health Policy (2014) described the promotion for older age groups [51]. It also mentioned that launching special oral health care education for older people and their caregiver/family is essential to preventing adverse effects of aging on oral health of older population. Similarly, Jamaica’s oral health policies mentioned that oral health education programmes need to be launched to promote older population’s oral health [49].

### B) Selection of scientific published literature relating to preventive and therapeutic interventions to ensure good oral health in the older population

**Fig. 1:**
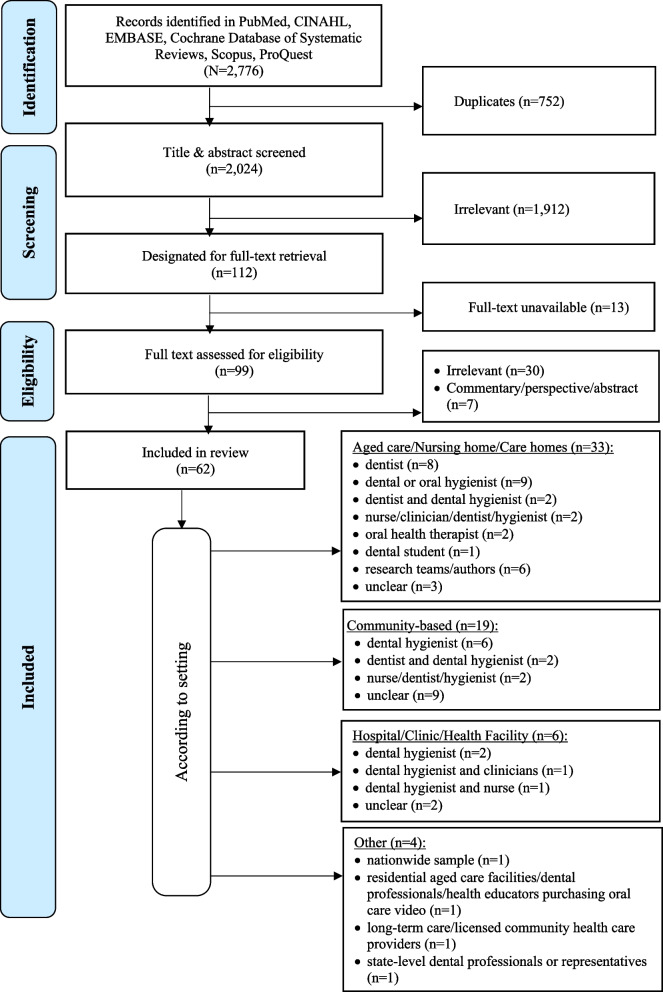
Study selection process (PRISMA-ScR) [33]

The systematic bibliographic database searches retrieved a total of 2776 records. After excluding 752 duplicates and another 1912 records due to ineligibility upon screening of the title and abstract, 112 records remained for full-text review. Thirteen citations were further excluded because the full text versions could not be readily accessed. Of the 99 full-texts, 62 met the inclusion criteria and were included in this scoping review (Fig. 1).

### Study characteristics

#### Study design

The characteristics of the 62 studies included in this review are presented in Table 1. The most common study design was interventional study (*n* = 57) followed by observational study (*n* = 4) and a comparative/review study (*n* = 1).

#### Geographical location

The majority of the included studies were conducted in HICs (*n* = 56), namely, the United States of America (*n* = 8), Australia (*n* = 7), Japan (*n* = 7), South Korea (*n* = 6), United Kingdom (*n* = 4), Canada (*n* = 3), Germany (*n* = 3), Netherlands (*n* = 2), Hong Kong (*n* = 2), Taiwan (*n* = 2), and others. Only six studies were from LMICs, namely India, Indonesia, Colombia, Iran, Brazil, and Thailand.

#### Study settings and number of participants

The majority of the studies were conducted in aged care facilities/ nursing homes/ long-term care institutions (*n* = 33/62), followed by community settings (*n* = 19/62) and hospitals/ clinics/ health care facilities (*n* = 6). In addition, two studies included nationwide samples, one aimed to assess change in dental service use after implementing the national health insurance covering dental services to older population whereas the other study assessed the usefulness of practical oral care video among the video purchasers. Moreover, a study was conducted among the service providers from long-term and community-licensed healthcare professionals. Furthermore, a qualitative study was conducted among the state level dental professionals or representatives. The sample size in the included studies varied widely, ranging between 12 and 17,264 (Table 2).

**Table 2:** Study characteristics (*N* = 62)

Authors (Year) Country: Setting [Ref.#]	Study Aims	Study Population	Inclusion Criteria
Intervention Studies			
Little et al. (1997) USA: Community based [53]	Effectiveness of group-based behaviour modification intervention on oral hygiene skills, adherence, and clinical outcomes	107 older adults I: 54 C: 53	Age 50–70 years, ≥ 18 teeth, no hepatitis B, diabetes, or immunodeficiency, not taking medications to affect the gingival health or requiring no prophylactic antibiotic premedication
Mojon et al. (1998) Switzerland: Long-term care facility [54]	Effectiveness of preventive oral health program	116 residents I:58 C:58	Age > 65 years
Simons et al. (2001) United Kingdom: Residential home [55]	Effectiveness of chlorhexidine acetate/xylitol gum on the plaque and gingival indices	111 residents	Age > 60 years, dentate, had not taken antibiotics within 4 weeks
Engfors et al. (2004) Sweden: Clinic based [56]	Clinical and radiologic performances of fixed implant-supported prostheses placed in edentulous elderly patients	248 patients I:133 C:115	Age ≥ 80 years, had implant surgery between January 1986 and August 1998. no bone grafts
Mariño et al. (2004) Australia: Community-based [57]	Effectiveness of oral health promotion program on the use of oral health services, oral health knowledge, attitudes, and practices	520 older adults	Age > 65 years, Italian or Greek background, attended senior citizens clubs
Wyatt et al. (2004) Canada: Long-term care [58]	Effectiveness of daily mouth rinse with neutral sodium fluoride or chlorhexidine gluconate in reducing the caries	116 residents	Residents having natural teeth; > 3-year life expectancy; ability to use a mouth rinse
Peltola et al. (2007) Finland Hospital [59]	Effectiveness of using electric toothbrush and interdental toothbrush	130 participants	Chronically ill older patients at last years of life in the hospital
Hakuta et al. (2008) Japan: Senior citizens’ centres [60]	Effectiveness of oral function promotion programme in oral health status and oral function	141 older adults I:79 C:62	Independent elderly attending senior citizens’ activity centres in the Tokyo metropolitan area
Baca et al. (2009) Spain Residential home [61]	Effectiveness of a chlorhexidine-thymol varnish on dental root caries	68 residents	Age 65 years, > 6 teeth, no serious disease, no intake of antibiotics within 2 weeks
Tan et al. (2010), Hong Kong: Nursing-home [62]	Effectiveness of four different methods in preventing new root caries	306 residents	Residents with > 5 teeth with exposed roots, no serious medical problems, basic self-care ability
Blinkhorn et al. (2011) Australia: Aged care facility [63]	Introduction of innovative nursing intervention to improve oral health	30 residents All qualified nur-ses at the ward	Residents with mental health problems
Tashiro et al. (2011) Japan: Nursing home [64]	Effectiveness of toothbrushing, tongue cleaning, chlorhexidine use	12 residents	Dependent residents w/self-brushing difficulty; > 10 teeth, no dentures
Mariño et al., (2013) Australia: Community based [65]	Effectiveness of theory-based oral health promotion intervention by a nondental peer educator	144 older adults I:74 C:70	Age ≥ 55 years, Italian club member, living independently
Van Der Putten et al. (2013) Netherlands: Care homes [66]	Effectiveness of ‘Oral health care Guideline for the Older population in Long term care institutions’ and a daily oral health care protocol	232 older adults	Residents possessing teeth or removable partial/complete dentures, physically suitable for examination & those expected to reside in care home for the entire 6 mos period. Inclusion criteria for care home include: 120–250 beds, somatic & psychogeriatric wards, ≤100 km from the center of The Netherlands
Zenthofer et al. (2013) Germany: Long-term care [67]	Effectiveness of professional cleaning of tooth and dentures	106 residents	Residents with care level 1 or no care level and not suffering from dementia or severe infectious diseases
Zhang et al. (2013) Hong Kong: Community [68]	Investigate synergetic use of SDF & OHE	266 older adults	Age 60–89 years, able to perform daily self-care, ≥ 5 teeth with exposed root surfaces, no life-threatening diseases
Bonwell et al. (2014) USA: Long-term care & community [69]	Interprofessional education (IPE) approach to educate direct health care providers	145 health service providers	Students & providers from long-term care and community-licensed health care professionals
Morino et al. (2014) Japan: Nursing-home [70]	Effectiveness of short-term oral health care on oral microbiological parameters	34 residents	Age > 64 years, > 1 tooth
Kammers et al. (2015) Brazil: Condominium for the elderly [71]	Effectiveness of individually adapted toothbrush handles in reducing of biofilm on dentures	16 residents	Residents in a good state of cognition, wearers of complete maxillary dentures
Khanagar et al. (2015) India: Institutionalized elderly [72]	Educating caregivers	322 residents from 7 elderly homes I:162, C:160	Residents willing to reside in the institution for up to 6 mos during intervention; > 10 natural teeth or dentures; cognitively & physically fit to undergo oral examination
Komulainen et al. (2015) Finland: Community [73]	Effect of oral health-promoting intervention	279 older adults	Age > 75 years, from Kuopio, Finland
Ohara et al. (2015) Japan: Community [74]	Effectiveness of oral health educational program on oral health and function	47 older adults	Age ≥ 65 years
Kim et al. (2016) South Korea: Long-term care [75]	Effectiveness of oral health promotion program	50 residents I:33 C:17	Age > 65 years, capable of communication and self-toothbrushing
Mariño et al. (2016) Australia: Community [76]	Evaluation of web-based oral health promotion programme	47 older adults	Age ≥ 55 years, from Whittlesea, functioning independently
Avellaneda et al. (2017) Colombia: Social protection centres [77]	Oral health education strategy: design & implementation	68 residents	Age > 60 years, good mental & physical health
Deutsch et al. (2017) Australia: Aged care facility [78]	Advanced oral health training to nurses to integrate individualised oral care plans	8 residents 4 staff (nurse/ assistants)	Early dementia residents Nurses with > 4 years of aged care nursing experience, no prior oral health interventions education
Ildarabadi et al. (2017) Iran: Nursing-home [79]	Effectiveness of oral health care program	101 residents I:46 C: 55 31 caregivers	Residents with no dental and medical interventions within 3 mos
Nihtila et al. (2017) Finland: Community [80]	Effectiveness of tailored preventive oral health intervention	269 older adults	Age > 75 years community-based home care clients from 3 communities in Eastern and Central Finland
Sakashita et al. (2017) Japan: Community [81]	Evaluation of program fostering a self-management ability	150 older adults	Age ≥ 60 years
Schwindling et al. (2017) Germany: Nursing home [82]	Effectiveness of oral health education and use of ultrasonic devices for denture cleaning	269 residents	Participants whose target variables could not be evaluated were excluded (e.g., edentulous residents without dentures)
Fjeld et al. (2018) Norway: Nursing home [83]	Longer term effect of tooth brush use as per residents’ own preference	204 residents	Those residents having 6 or more natural teeth
Janssens et al. (2018) Belgium: Nursing home [84]	Impact of an oral healthcare program in nursing homes on the initial treatment backlog and residents’ oral health stability	381 residents	Residents who visited the mobile dental clinic for a first consultation between October 2010 (i.e., when Gerodent started) and April 2012.
Lavigne et al. (2018) Canada: Nursing home [85]	Effectiveness of twice-daily use of rotating-oscillating power toothbrush in periodontal inflammation	59 residents	Those residents having 6 or more natural teeth
Marchini et al. (2018) USA: Nursing home [86]	Evaluate feasibility to test clinical and microbiological effectiveness of a nursing facility	81 residents	All residents
Seleskog et al. (2018) Sweden: Nursing home [87]	Effectiveness of oral health care education program	37 residents (I:15, C: 22) 53 staff (I:23, C = 30)	Residents
Berniyanti et al. (2019) Indonesia: Nursing home [88]	Effectiveness of counselling and training on toothbrushing for full/partial edentulous teeth	12 residents	Older adults from the selected nursing homes
Iwao et al. (2019) Japan: Community [89]	Effectiveness of oral health prevention program (3 month)	43 older adults	Age ≥ 65 years Exclusions: on long-term support, stroke related motor paralysis
Keyong et al. (2019) Thailand: Community [90]	Effectiveness of an oral health promotion program	162 older adults	Age 60–74, ≥ 6 teeth, no disability
Leon et al. (2019) Chile: Community [91]	Effectiveness of toothbrushing with 5000 ppm versus 1450 ppm fluoridated dentifrice	345 older adults	Age ≥ 60 years, from areas with fluoridated water (0.7 ppm F), independently-living
Laurence et al. (2019) USA: Long-term care [92]	Effectiveness of checklist for oral care	32 residents I:19 C:13	English speaking patients, > 4 teeth
Saleem et al. (2019) Japan: Hospital [93]	Effectiveness of lip trainer device (Group P) & sonic toothbrush (Group S). Control group (Group C)	39 patients Group P:13 Group S:13 Group C:13)	≥60 years, ≥20 teeth, with periodontal disease referred to hospital between April 2013 & Dec 2016
Tellez et al. (2019) USA: Dental clinics [94]	Examining treatment fidelity of an individual-based MI intervention	60 patients	Age ≥ 55 years, attending dental clinics appointment
Tellez et al. (2020) USA: Attending the dental clinics [95]	Efficacy of individualised ‘Motivational Interviewing’ approach to oral health education	180 patients	Age ≥ 55 years, fluent in English, with scheduled dental appointment
Ting et al. (2019) Taiwan: Community [96]	Effectiveness of an oral health educational programme	539 older adults	Age ≥ 65 years, can perform oral function exercises
Johansson et al. (2020) Sweden: Nursing home [97]	Feasibility of oral health coaching programme	33 staff (I: 24 and C: 9) and 46 residents (I: 30 and C: 16)	Staff and all residents
Konstantopoulou et al. (2020) Greece: Nursing home [98]	Design, implement, and evaluate oral health education program for nursing home caregivers	55 caregivers (I:28, and C:27)	Caregivers
Lee et al. (2020) South Korea: Nursing home [99]	Effectiveness of oral health care program	135 residents (45 per group)	Age ≥ 65 years not received any dental care within 6 mos.
Lee et al., (2020) South Korea: Community [100]	Effects of oral health education programme utilising a workbook	120 older adults (I:40, 40 and C:40)	Age ≥ 65 years, able to read Korean, able to walk/move
Ho et al., (2021) The Netherlands: Community [101]	Early recognition of decreased oral health status and establishing a need for interprofessional care	407 older adults and 50 healthcare professionals	Community-dwelling frail older people Healthcare professionals from general practice, a dental practice, home care organization
Ki et al., (2021) South Korea: Community [102]	Effectiveness of oral health education using a mobile app (OHEMA) on oral health & SWAL-QoL	46 older adults (I:24 and C:22)	Able to communicate without linguistic, auditory or visual impairment, normal cognitive ability
Lee et al., (2021) South Korea: Senior welfare centre [103]	Effectiveness of oral health education programme	90 older adults	Age > 65 years, able to read and understand Korean
Northridge et al., (2021) USA: Community [104]	Acceptability of a community health worker intervention on oral health	74 older adults	Older Chinese Americans (Chinese immigrants)
Peroz & Klein (2021) Germany: Residential/ retirement home [105]	Investigate influence of quarterly professional dental hygiene treatment	160 (Home A: 99 and Home B: 61)	All residents
Patel et al. (2021) UK: Residential/ nur-sing care homes [106]	Introducing risk-based preventative OH program	49 residents	Age > 65 years
Pawluk et al. (2021) Canada: Residential care facility [107]	Impact of online oral health education module on personal support workers’know-ledge and beliefs	109 (88 residents for Quant; 21for Qual data collection)	All personal support workers
Sun et al., (2021) Taiwan: Community [108]	Effectiveness of easy-to-read health education materials to improvement oral heath literacy	129 older adults (I:72 and C:57)	Age ≥ 60 years, communicating in Mandarin Chinese/Taiwanese Hokkien
Wanyonyi et al., (2021) UK: Primary dental care centre [109]	Comparison of acceptability and perceived helpfulness of an e-oral health intervention	150 patients (I:76 and C:74)	Age ≥ 65 years dentate; communicate in English
Observational study			
Strayer (1991) USA: State level [110]	Explore existing and possible future oral health programs	48 participants	State level dental directors or dental program managers (If none: health department administrators
Chalmers et al. (2005) Australia: Mixed samples (RACFs/dental professionals/ health educators) [111]	Usefulness and appropriateness of the Practical Oral Care Video	294 video purchasers	Practical Oral Care video purchasers
McAnulla et al. (2018) UK: Nursing home [112]	Assess caregivers’ awareness & knowledge on oral health care	N/A	N/A
Lee et al., (2021) South Korea: Nation- wide [113]	Assess the denture procedure among the older adults	17,264 older adults	Age ≥ 65 years, under National health insurance
Comparative/ Review Study			
Tynan et al. (2018) Australia: Aged care facilities [114]	Impact and experience of integrated approach to oral health	252 residents (111 in integrated model (IM) and 141 without IM)	All residents

*C* Control group participants, *I *Intervention group participants, *SDF *Silver diamine fluoride, *SWAL-QoL *Swallowing related quality of life

### Types of intervention/program

The majority of studies included interventions for prevention and management of oral health conditions among older populations. A wide range of oral care interventions/programs were adopted and the target groups included both the older people (and family members) and care providers. The most common intervention (*n* = 24) was to raise awareness on oral health through courses, trainings or educational sessions and distribution of Information Education and Communication (IEC). The educational intervention was provided using a wide range of methods, such as, face to face interview, group sessions, practical oral care videos and text messages via phone, and smartphone applications. Similarly, some studies (*n* = 6) also involved practical sessions and demonstrations, along with the educational session. Two studies (*n* = 2) applied motivational interviewing techniques to educate on oral health and assess the treatment fidelity of motivational interviewing. Furthermore, three studies (*n* = 3) adopted mobile phone-based text messages and two studies (*n* = 2) involved web-based online oral health education and promotion programs to improve knowledge, attitudes, and self-care practices aimed at attaining and maintaining oral hygiene and health.

Several studies also involved interventions to improve oral health status, such as improving salivary flow and oral wetness. The interventions used in these studies included application of silver diamine fluoride (*n* = 2), toothbrushing with fluoride toothpaste (*n* = 6), fluoride varnish (*n* = 2), chlorhexidine rinsing(*n* = 6), ultrasonic devices for denture cleaning (*n* = 1), and lip muscles trainer device and sonic toothbrush (*n* = 1).

In addition, a qualitative study among dental professionals explored understanding of the ongoing oral health programs for older adults. Similarly, one retrospective study assessed the effectiveness of 5-year clinical and radiologic performance of fixed implant-supported prostheses placed in edentulous older people.

### Involvement of dental professionals

The most common dental professionals providing intervention were dental/oral hygienists (*n* = 17), followed by dentists (*n* = 8), both hygienists and dentists (*n* = 4), oral health therapists (*n* = 2), dental nurse (*n* = 1), and dental students (*n* = 1). Five studies used a combination of dental and non-dental professionals, namely nurses, physicians, dieticians, pathologists, and pharmacists, whereas one study involved community health workers providing the oral health intervention. Information regarding who provided the interventions was not clearly reported in 24 studies.

### Results from published literature

The results of the studies (Table 3) are reported as per the study settings.

**Table 3:** Key study findings (*N* = 62)

Authors (Year) [Ref.#]	Intervention	Duration	Interven-tionist	Key Findings
Intervention Studies				
Little et al., (1997) [53]	Five 90-minute oral hygiene classes to intervention group. Control group: usual care	4 mos	Dental hygienist	Brushing frequency & skills margi-nally better in treatment group, but mean % sites with plaque, gingival bleeding & bleeding after probing were similar in both groups
Mojon et al. (1998) [54]	Educational sessions (Oral hygiene courses) to healthcare providers & regular calls to residents. Control group treated by dentist with mobile equipment by request	18 mos	Dental hygienist	
Simons et al. (2001) [55]	Chlorhexidine acetate/ xylitol gum. Control group: usual care	12 mos	Not clear	Improved plaque & gingival scores with chlorhexidine/ xylitol gum
Engfors et al. (2004) [56]	5-year clinical & radiologic performances of fixed implant-supported prostheses (Retrospective)	Retrospective study (5 years)	Not clear	The result of Implant treatment among the older adults & younger age group were comparable.
Mariño et al. (2004) [57]	ORHIS implemented (incl. Access to services & referrals). Control group: usual care	12 mos	Not clear	ORHIS approach significantly improved oral health attitudes, knowledge & behaviours on oral care
Wyatt et al. (2004) [58]	Application of 15 ml of either 0.2% neutral NaF or 0.12% chlorhexidine gluconate. Control group: placebo	24 mos	Dentist	Reduced incidence of caries
Peltola et al. (2007) [59]	Group A: tooth cleaning (electric toothbrushes & interdental brushes) by dental hygiene students; group B: same services by dental hygienist trained nurses. Control usual care.	11 mos	Dental hygienist (students) Nurse	Intervention group B performed best in terms of improved denture & dental hygiene
Hakuta et al. (2008) [60]	Intervention participants received educational sessions & encourage-ment for facial muscle & tongue exercise, & salivary gland massages. Control gtoup received usual care	3 mos	Dental hygienist	Reduced tongue coating scores, organoleptic score for oral mal-odours & food debris in oral cavity. Improved tongue dryness, lip movement & clearer pronunciation
Baca et al. (2009) [61]	Application of a chlorhexidine-thymol varnish. Control group = Placebo group	12 mos	Dentist	Reduced incidence of root caries lesions
Tan et al. (2010) [62]	4 different methods: i) individualized OHI; ii) OHI & 1% chlorhexidine varnish every 3 mos; iii) OHI & 5% sodium fluoride varnish every 3 mos; iv) OHI & annual 38% SDF solution	36 mos	Unclear	SDF solution, sodium fluoride varnish & chlorhexidine varnish more effective in preventing new root caries lesions than oral health instructions alone
Blinkhorn et al. (2011) [63]	Design & produce oral hygiene trolley, develop protocol & educate staff. Comparison between baseline & endline	12 mos	Nurse Dentist Dietician	Reduced plaque & gingivitis scores & pocket depths
Tashiro et al. (2011) [64]	Pre-post-intervention in 3 groups i) oral cleaning by toothbrushing alone; ii) tongue coat removal using sponge brush; iii) wiping oral mucosa with gargling solution containing chlorhexidine gluconate	5 conse-cutive days every 3 weeks	Dentist Dental hygienist	Improved oral malodour; decreased plaque & gingival index scores
Mariño et al., (2013) [65]	A series of oral health seminars & 4 supervised brushing sessions at club premises. Control group: usual care	6 mos	Not clear	Improved gingival status & self-efficacy, but no effect regarding dental plaque
Van Der Putten et al., (2013) [66]	The supervised “oral health care Guideline for older people in long-term care institutions” (OGOLI) was introduced to the intervention arm. Control group: usual care	6 mos	Dental hygienist	Improved dental plaque scores
Zenthofer et al. (2013) [67]	Professional cleaning of teeth & dentures. Control group: usual care	3 mos	Dentist	Improved dental hygiene, plaque & gingival index scores
Zhang et al. (2013) [68]	2 groups: i) OHI & SDF; ii) OHI & SDF, plus OHE. Control group: OHI	24 mos	Dentist Dental hygienist	Intervention group had greater num-ber of active root caries surfaces that became arrested than control group
Bonwell et al. (2014) [69]	Two 45-mintes in-service training sessions with demonstrations to health care providers. Pre- & post-test comparison	3 mos interval	Periodon-tist, Oral pathologist, Pharmacist, Dietitian Occupatio-nal therapist	~ 80% of the 145 participants indicated they would make a change in patient care
Morino et al. (2014) [70]	Professional oral health care & toothbrushing. Control group: usual care	5 mos	Dental hygienists	Improved dental plaque score
Kammers et al. (2015) [71]	Application of adapted toothbrush handles. Control group: conventional toothbrush	0.75 mos	Dentist	Reduction in biofilm coverage among those using adapted toothbrush handles
Khanagar et al. (2015) [72]	Educational sessions (through PowerPoint). Control group: usual care	6 mos	Not clear	Improved oral health knowledge of caregivers & reduced scores for plaque, debris, denture plaque & denture stomatitis
Komulainen et al. (2015) [73]	Intervention group received individually tailored personal guidance in dental & denture hygiene. Control group: usual care	24 mos	Dentist Dental hygienist	Improved oral health among intervention group compared to control
Ohara et al., (2015) [74]	Intervention group received OHE programs. Control group received pamphlets describing only general information about oral health	3 mos	Dental hygienist	Improved resting salivation, second & third swallowing times as well as taste sensitivity for bitterness
Kim et al. (2016) [75]	Oral health promotion program (Combined Watanabe method & oral functional exercise). Control group: usual care	Not clear	Not clear	Improved oral hygiene & oral function scores
Mariño et al. (2016) [76]	Pre- & post-ORHIS model via com-puter interactive presentations; no direct role of oral health professionals	12 mos	Not clear	Significantly improved oral health attitudes, knowledge & self-efficacy & self-reported oral hygiene practices
Avellaneda et al. (2017) [77]	Educational sessions & demon-strations on oral care & tooth brushing techniques. Comparison between baseline & endline	6 mos	Dental students assessed by panel of experts	Reduced plaque & gingival scores
Deutsch et al. (2017) [78]	Educational sessions & training to nurses in oral assessments & saliva testing & develop care plan. Com-parison between baseline & endline	10 weeks	Oral health therapists	Enhanced competencies of nurses so they could choose the appropriate intervention similar to oral health therapists
Ildarabadi et al. (2017) [79]	Educational session through training. Control group: usual care	2 mos	Not clear	Improved oral health status
Nihtila et al. (2017) [80]	Oral/written dental hygiene instruc-tions & cleaning of oral mucosa provided to participants/caregivers/ nurses. Control group: usual care	6 mos	Dental hygienist	Improved denture hygiene & reduced number of plaque-covered teeth
Sakashita et al. (2017) [81]	Knowledge, skill & experience sharing on self-care. Private consul-tation to manage condition through oral examination. Baseline, 3- & 6-mos intervention comparisons	6 mos	Nurse, Dentist Dental hygienist	Improved use of dental floss & interdental brushing, Community Periodontal Index scores & deposits of plaque & oral & physical quality of life
Schwindling et al. (2017) [82]	Educational sessions & practical training (different types of prosthe-tic restorations) for care-givers. Control group: no intervention	12 mos	Dentist	Improved plaque & gingival bleeding scores
Fjeld et al. (2018) [83]	New toothbrush by own preference & application of 1450 ppm NaF toothpaste	12 mos	Dentist, Dental hygienist	No differences in plaque scores between manual and electric toothbrush
Janssens et al. (2018) [84]	Educational sessions & implemen-tation of oral healthcare guideline. Comparison between baseline & FU	22.5 mos (Mean)	Dentist	Reduced proportion of oral health incident treatment need
Lavigne et al. (2018) [85]	Twice-daily use of a rotating-oscillating power toothbrush. Control group: usual care	1.5 mos	Dental hygienist	Reduced periodontal inflammation including reduction in bleeding
Marchini et al. (2018) [86]	Group 1) Educational session; 2) educational session plus application of chlorhexidine varnish. Control group: usual oral hygiene practice	6 mos	Dental hygienist	No clinical differences recorded in clinical or microbiological outcomes
Seleskog et al. (2018) [87]	Individualized guidance & support for each resident. Control group: usual care	3 mos	Dental hygienist	Improved plaque levels in older adults; enhanced capacity of nursing staff to perform proper oral care
Berniyanti et al., (2019) [88]	Counselling about dental & oral health materials & ways of brushing toothless jaws	Not clear	Not clear	Average improvement of knowledge & application of toothbrushing method
Iwao et al. (2019) [89]	Educational sessions & demonstra-tions on physical exercise, oral health & nutritional guidance. Com-parison between baseline & endline	3 mos	Dental hygienist	This intervention may contribute to healthy aging in older people
Keyong et al. (2019) [90]	OHE & demonstration of oral hygiene & denture cleaning, provi-sion of toothbrush & fluoride tooth-paste. Control group: those who did not practice tooth brushing	6 mos	Dental nurses	Improved oral health perception, lower plaque & gingival & inflammation scores & less clinical attachment loss
Leon et al. (2019) [91]	Application of 5000 ppm NaF-dentifrice. Control group: 1450 ppm NaF-dentifrice	24 mos	N/A	5000 ppm F-dentifrice more effective than conventional dentifrice in preventing & arresting RCLs
Laurence et al. (2019) [92]	Educational sessions for nursing staff. Control group: those not receiving intervention	2 mos	Dentist	Improved plaque scores
Saleem et al. (2019) [93]	Provided lip trainer device to one group, sonic toothbrush to other group & others as control	6 mos	Clinician Dental hygienist	Lip training device improved salivary flow rates & oral wettability. But less improvement observed among those using a sonic electric toothbrush
Tellez et al. (2019) [94]	Motivational interview sessions	Not clear	Dental hygienist	Basic or greater proficiency achieved in improving oral health
Tellez et al. (2019) [95]	Motivational interview sessions. Compared with traditional OHE group & control group: usual care	12 mos	Dental hygienist	Improved oral health self-efficacy (SE) & OHRQL in intervention groups. No change in control group
Ting et al., (2019) [96]	Brief OHE program incl. Education materials. Comparison between pre- & post-test	8 mos	Not clear	Improved GOAHI score. Significant differences for RSST, ODT & CET
Johansson et al. (2020) [97]	Educational sessions for nursing staff. Demonstration sessions to each resident. Control group: usual care	3 mos	Dental hygienist	Nursing staff moderate/high, residents good/acceptable oral health & oral health care-related beliefs at baseline, which was maintained
Konstantopoulou et al. (2020) [98]	Educational sessions. Control group: usual care	2 mos	Not clear	Improved knowledge & attitude
Lee et al. (2020) [99]	Professional oral health care program. Control group: usual care	3 mos	Dental hygienist	Reduced tongue coating, plaque & gingival scores
Lee et al., (2020) [100]	Oral health education only to 1 group & other group had additional access to contents of oral health education. Control group received usual care	1.25 mos	Not clear	Increased oral health knowledge oral health recognition among the intervention group
Ho et al., (2021) [101]	Oral health education consisting oral health care for frail older adults, practical sessions on daily oral hygiene care	12 mos	Geriatric dentist Dental hygienist Geria-trician	Increased oral health awareness amongst health care professionals
Ki et al., (2021) [102]	Intervention group received 4 videos on oral exercise, intraoral & extraoral massage & oral hygiene on brushing & denture care methods along with a workbook & a poster. Control group: usual care	1.5 mos	Not clear	Improved SWAL-QoL, increased tongue pressure & reduced oral dryness
Lee et al., (2021) [103]	Oral health education via smartphone app developed in this study & via PowerPoint lectures to non-app users. Control group: usual care	1.25 mo	Not clear	Improved oral health knowledge & perception & reduced dental plaque & tongue coating scores amongsmartphone users
Northridge et al., (2021) [104]	Training on proper toothbrushing & flossing techniques. Encouraged regular dental visits & brushing with fluoride toothpaste & addressed any expressed concerns. Pre- & post-test comparison	1 mo	Community health worker	> 98% participants strongly agreed/ agreed that CHWs helped them improve taking care of their oral health, answered participants’ questions/concerns & in-person demonstrations were effective to improve oral health
Peroz & Klein (2021) [105]	Education sessions & training for staff & oral hygiene treatment for residents. Control group: usual care	1 year	Dentist	Oral parameters (pocket depth, denture hygiene, mucosal alterations) may be positively influenced
Patel et al. (2021) [106]	Fluoride toothpaste use & quarterly fluoride varnish. Comparison between baseline & endline	12 mos	Trained clinician Dentist Hygienist	Prevented root caries
Pawluk et al. (2021) [107]	Educational sessions (Online modules). Comparison between pre- & post-test	Not clear	Dental hygienist	Limited impact on Personal Support Workers’ knowledge & beliefs regarding resident oral health care
Sun et al., (2021) [108]	Easy-to-read health education materials with PowerPoint slides. Control group received general text materials on oral health to read	30 minutes after session	Not clear	Improved oral health literacy
Wanyoni et al., (2021) [109]	Text messages on dental & oral care to intervention group. Control group: received leaflet on dental & oral care	2.5 mos	Not clear	89% of participants in text arm would recommend the intervention versus 68.2% in the leaflet arm
Observational Studies				
Strayer (1991) [110]	The intervention program reduced *Streptococcus mutans* colonisation & caries prevalence	N/A	N/A	Oral health programs for the elderly reported in 30 states (63% of respon-dents). Perceived or documented need for oral health programs for the elderly & lobbying by local advocacy groups were instrumental in implementing or planning such programs
Chalmers et al. (2005) [111]	Practical Oral Care video	N/A	N/A	Intervention improved awareness about oral health issues
McAnulla et al., (2018) [112]	Poster containing instructions on maintaining oral care & provision of box containing oral hygiene resources	N/A	N/A	Improved awareness of & attention to the oral care of older adults
Lee et al., (2021) [113]	Implementation of National Health Insurance Coverage of Dentures for the elderly	2011–2013	N/A	Increased denture procedures for older adults and low-income & medical aid beneficiaries
Comparative/ Review Study				
Tynan et al. (2018) [114]	Integrated oral health program (screening, education & referrals). Control group: facilities without integrated program	N/A	Oral health therapist	Improved compliance with Australian Aged Care Quality Accreditation Standards

*CET* Cheek expanding test, *CHW *Community health worker, *FU *Follow-up, *mo(s) *Month(s), *GOAHI *Geriatric/general oral health assessment index, *incl. *Inclusive, *NaF *Sodium fluoride, *ODT *Oral diadochokinesia test, *OHE *Oral health education, *OHI *Oral hygiene instructions, *OHRQL *Oral health-related quality of life, *ORHIS *Oral health information seminars/sheets, *RCL(s) *Root caries lesion(s), *RSST *Repetitive saliva swallowing test, *SDF* Silver diamine fluoride, *SWAL-QoL* Swallowing-related quality of life.

#### Nursing homes/RACFs/long-term care settings

The major interventions conducted in the nursing home, RACFs, or long-term care setting were oral health education and promotion programs for the older residents that included hands-on guidance and support in oral care [87], toothbrushing [64, 85], oral hygiene and denture care [72, 77, 82], and oral functional exercises [75]. Such interventions reported significant reduction in plaque and gingival index and improved oral health related quality of life of these older residents.

Two studies focused on interventions for the caretakers of older residents that included educational sessions and demonstrations on oral hygiene and denture care [92, 98]. Such intervention reduced the plaque and gingival indexes in the older residents and improved knowledge and attitudes on oral health among their caretakers.

Four studies enrolled both the older residents and staff consisting of care givers and nurses [63, 78, 79, 87]. Interventions included instruction on oral health care and daily oral hygiene routines [87], training on oral assessments and saliva testing [78], installation of an oral hygiene trolley, protocol for oral hygiene, and provision of education [63] and educational sessions and training on oral care [79]. The results were improved oral health status of the older residents and enhanced oral health competencies of the nurses and caregivers [82].

Seven studies involved clinical interventions among older adults through mobilizing their care givers and nursing staff. The interventions included tongue cleaning and wiping the oral mucosa with a sponge soaked in chlorhexidine [64]; professional oral health care programs involving dentists and dental hygienists regularly monitoring and managing the oral health of older residents [70, 115]; application of chlorhexidine varnish [58, 61, 62], and fluoride [106]. The results of such interventions were significant declines in plaque and gingival indexes, reduced opportunistic infections, and prevention of incident root caries in these older residents.

#### Hospitals/clinics/other health facilities

Of six studies, four studies included only older people attending for care, with the remaining two studies also included other age groups. A wide range of oral health interventions were used in these studies, including face-to-face motivational interview sessions on oral health [94, 95], application of a lip muscle trainer device and sonic toothbrush [93], electric toothbrush and interdental brushes [59], and text messages on tooth brushing, flossing, fluoride use, and denture cleaning [109]. These interventions resulted in improved oral health outcomes, such as improvement in the salivary flow rate, oral wetness, and denture and oral hygiene.

A retrospective review of fixed implant-supported prostheses comparing the clinical performances among edentulous older patients and younger patients reported the clinical performances were similar between the groups [56]. However, cleaning problems; mucositis; and tongue, lip, and cheek biting were more common among the older compared to younger patients. A qualitative study was conducted with state level dental professionals, managers, or representatives [110] The study reported that the majority of oral health programs were focused on institutionalized patients and their caregivers; and that oral healthcare needs of older adults have been realized and can be best addressed by coordinating or lobbying with local advocacy groups.

#### Community-based settings

The interventions in community-based settings were mostly directed to independent older adults without physical limitations. The interventions on this setting were largely focused on organizing seminars, education, and demonstration sessions on oral health and hygiene [53, 57, 68, 76, 80, 89]; instruction in interdental flossing, and tooth brushing [65, 81, 90, 104]; denture and oral mucosa cleaning [80]; application of fluoride [73], silver diamine fluoride [68], and fluoride dentifrice [91]; and videos on tooth brushing and denture care methods [102]. In addition, oral function promotion programmes, including knowledge on oral health and encouragement of facial muscle and tongue exercises and salivary gland massage, were conducted [60, 74, 96]. Such interventions improved swallowing; reduced plaque index scores, gingival inflammation, oral malodour and oral dryness; and prevented root caries. Moreover, knowledge level and attitude and self-care behaviours regarding oral health also improved among these older adults.

A study from The Netherlands evaluated the public project “Don’t forget the mouth!” (DFTM project) involving healthcare providers from general medical practices, general dental practices, and home care organizations [101]. The health care providers participating in the DFTM project were provided with theoretical and practical sessions on oral health being associated with general health; oral health status of frail older adults; familiarization with oral health screening and referral tools. This study found that the DFTM project was effective in increasing the oral health awareness among the health care providers and hence in improving the oral health of the frail older adults. Nonetheless, several barriers exist with large-scale implementation, such as poor physical access to and lack of sufficient numbers of oral care providers and financial considerations [101].

## Discussion

The health burden and disease conditions in the older populations are likely to increase significantly with poor oral health status, if no appropriate actions are taken [1, 2, 11]. Efforts to save teeth and maintain good oral health, therefore, are crucial in geriatric populations. In this context, this scoping review explored (“scoped”) the existing evidence regarding oral health care for older population, namely policies/guidelines and interventions.

### Polices and strategies to promote oral health

This study identified only 17 policies specifically mentioned about oral health needs of the elderly. These polices primarily focused on access to oral health care services for older populations, as well as educating health professionals on the importance of oral health care and the relationship of oral health with overall health among the older population. Oral health policies that mentioned and prioritised oral health care for older people were from mainly from high income countries (HICs). Few policies, namely from Ireland [37], New Zealand [41] and Australia [47] and recognised the older population as a “vulnerable” group and highlighted their need for specific care models that could assist in accessing and navigating oral health care services. Furthermore, a need to include oral health risk assessment for all older people and development of an oral health care plan for those entering care facilities were also emphasized in these polices [47] [41]. It was emphasised that developing similar plans can potentially result in positive outcomes in relation to oral health status of older people [41]. The policies also emphasised the need for regular dental visits, oral prophylaxis (dental cleaning), and fluoride application in improving and maintaining good oral health of older populations [49]. These are considered important preventive measures to decrease the incidence of dental decay and periodontitis to prevent loss of teeth among older people [116].

Since access to dental care is often challenging for geriatric populations, particularly the institutionalised, there is an urgent need to develop policies and strategies that include measures to facilitate regular dental check-ups for this vulnerable group. In the absence of policies, it is challenging to address social determinants of oral health inequalities [117]. As such, clinicians may face difficulties in making decisions in the absence of clear guidelines [118], and prioritise integration of oral health care as a needed part of healthy ageing. This is reflected in the findings of the WHO survey (2017–2018) which showed that only 20% of the 101 countries surveyed, reported having programmes for oral health of older people, and this was even found less in low-income countries (4.8%) [119]. Overall, it is evident that there is currently limited focus and support for oral health of older people at the government level [119], which highlights the responsibility of the countries to formulate geriatric oral health polices and guidelines for prevention and management of oral diseases in older people.

In 2021, the 74th World Health Assembly (WHA74) approved the WHO resolution on oral health [120], recognising the global burden of oral diseases and their associations with systemic conditions, and urged all countries to address shared risk factors, enhance the professional capacity of oral health professionals, and include oral health in universal health coverage (UHC) benefit packages. The Global Strategy on Oral Health pointed out that the negative impact of oral health problems accumulate over time and have complex consequences in later life, particularly in relation to other NCDs [121] and the need of locally tailored and age-appropriate oral health strategies integrated within relevant health programmes across the life course [121]. This is even more important in the context of low income countries, since oral health may not be a priority for people in these regions due to other health priorities, so dental problems are often left untreated [119]. Furthermore, it is also reported that there is less emphasis on primary prevention of oral diseases and limited access to oral health care in developing countries [119]. Thus, these aspects need to be taken into consideration while developing geriatric-specific oral health care policies and strategies.

### Preventive measures to improve oral health

The studies included in this review used a range of interventions in promoting oral health among geriatric populations. They included educational sessions and demonstrations to older adults and their caregivers on oral hygiene and denture care [72, 77, 82] and tooth brushing [64]. Some studies also used one-to-one and face-to-face motivational interview sessions on oral health [94, 95]. Such programs were reported to improve awareness about the importance of oral health and oral/denture hygiene of older populations, resulting in a decrease in plaque and gingival index scores [80], and ultimately improving oral health related quality of life [75, 81, 94, 95]. Since the compliance with the recommended annual dental check-ups and good home oral hygiene behaviours (brushing twice and floss/clean dentures daily) is reported to be lower among older residents [122], increasing awareness about the importance of oral health and oral health problems and their association with systemic diseases could be an effective strategy to improve compliance with the recommended preventive behaviours. Studies report that people who are better informed about the risks and consequences of poor oral health are more likely to engage in positive health behaviours [123]. Therefore, health care providers should be motivated to play a proactive role to provide oral health education and motivation to the older patients to perform self-care oral hygiene, as these are effective to reduce the risks of oral diseases [124, 125].

The studies included in this review also reported a range of preventative and therapeutic measures that oral health care professionals employed to improve oral health outcomes of older population. These included tongue cleaning with a sponge [64], wiping oral mucous with a sponge brush soaked in chlorohexidine [64], application of chlorhexidine varnish [58, 61, 62], fluoride (toothpaste and or varnish) [73, 106], fluoride varnish [62], fluoride dentifrice (5000 ppm) [91], silver diamine fluoride [68], and xylitol or chlorhexidine [73], facial muscle and tongue exercise and salivary gland massages [60] including use of lip trainer device to perform facial mimetic muscle training [74, 93, 96], and scaling and cleaning the teeth’s crown and root surfaces [73].

It is widely known that decreased salivary flow (hyposalivation) is commonly found in older persons due to general pathologies and especially to the medications taken, which leads to difficulties in swallowing (dysphagia) and chewing (mastication) [3–5]. Furthermore, hyposalivation can lead to greater incidence of coronal and root caries and periodontitis [6, 7], dental diseases that are very prevalent in older populations [8, 9]. The studies included in this review reported interventions, such as exercise of facial muscle and tongue to be effective in improving salivary flow rate [60, 74, 93, 96]. A recent meta-analysis (*n* = 18 studies) explored the effectiveness of oral exercise in improving the masticatory function among people ages 18 years and older and reported that resistance exercises, such as chewing or clenching, were the most beneficial exercises to improve the bite force, while simple oral exercises was not found to have any significant effect [126]. The Japanese study included in this review used a lip trainer device for muscle training and found this to be effective in improving salivary flow and dry mouth [93]. However, it is also important to consider that mastication is a complicated process involving the movements of lips, jaw, tongue, cheek, soft palate and masticatory muscles, therefore, it may require more efforts than just the oral exercise for improvement [126].

The studies included in this review reported the benefits of application of antimicrobial agents or varnishes, such as fluoride, sliver diamine fluoride, chlorhexidine varnish, or fluoride and chlorhexidine combined, in the prevention of root caries. However, results regarding effectiveness of application of different antimicrobial agents reported in various RCTs/systematic reviews are inconsistent. A systematic review (*n* = 6 studies) looking at the effect of chlorhexidine varnish found little or no additional effect to professional cleaning and good home oral hygiene. Nonetheless, the meta-analysis of only three studies showed benefit for high-risk patients, such as the elderly with dry mouth [127]. Similarly, results from a systematic review of RCTs (*n* = 3) supports the effectiveness of sliver diamine fluoride in prevention of root caries in older population [128].

The studies also included interventions involving non-dental care professionals, mostly nurses and public health professionals, and included education, training or meetings [63, 82, 97, 101], daily use of checklist for oral care [92], observation delivery of integrated oral health program [114], and development of an oral health protocol [63] to facilitate integration of oral health, such as education, oral assessments and referrals. These interventions were found to improve oral health care related knowledge and attitudes, and competencies [63, 82, 97, 101, 114], as nurses were positive about accepting oral hygiene into their daily routine care [63], This is consistent with the results reported in systematic reviews [129, 130]. Such practices, as reported in the Australian study, also resulted in improving oral health outcomes among older people, including reductions in plaque scores, gingivitis, and pocket depths [63].

Our study did not find any studies involving only dental care professionals. It is commonly reported that there is a gap in the knowledge and confidence among oral health care professionals (OHCP) regarding provision of oral care for old people [131], as older people are often vulnerable due to the weakening physical ability and cognition, and comorbidities. Furthermore, a systematic review reported lack of adequate equipment and space for oral treatment in care homes, as well as education and guidelines to manage oral health problems as commonly reported barriers among OHCPs [132]. These are perceived barriers for non-dental care professionals as well [133]. This warrants a need for education, training, and hands-on sessions for OHCPs as dental care needs and problems in geriatric population are often complex [134]. Providing this education and practice in undergraduate courses across disciplines is desirable to improve the oral health knowledge and competency of new graduates. Furthermore, providing dental treatment for people with impaired cognitive function make geriatric dental care even more challenging [134]. All these complexities together demand a shift in the oral health care model, as there is a need for multidisciplinary approach of oral health promotion in older people that involves nurses and other health professionals, such as GPs and dietitians, and the integration of oral health into overall health management of older people. As evidenced in several studies, nurses can provide support to dentists and can also independently play a proactive role in promoting goof oral health among older people in RACF, provided that they are supported with education and resources [28].

Overall, the findings of this review suggest that there is lack of policies and guidelines regarding oral health care for older people. A wide range of interventions are used to promote oral health care of older populations, however, there is a lack of information and research regarding the most effective treatments and evidenced-based guidelines for providing oral health care to older people.

### Implication of the findings

The results of this review have several implications for policy makers, geriatric care providers and oral health care professionals. The national oral health policies should focus and priorities to address oral health needs and care of geriatric population and prioritise to develop guidelines relating to oral health care of older people. Geriatric care providers could play a more active role in promoting oral health among their patients, such as in educating patients about their increased risk for oral health complications and advise them for regular dental visits. Oral health care professionals also should look for opportunities to train them to address oral health concerns of older people. Specific guidelines need to be developed to assist both, oral health care professionals and geriatric care providers in promoting oral health care in older people.

### Limitation

The scoping review undertaken has few limitations. The review did not look for policies published in other than English languages and as well as for unpublished articles in the interventions/programs and hence there is a possibility that this review may not have retrieved all policies/studies in this area. Similarly, there is a possibility that the search may have missed to capture articles due to the search in particular databases (six) and the search strategies. Lastly, although quality assessments of the studies were not undertaken, this additional information may have useful for synthesising the evidence.

## Conclusion

The findings of this review suggest that educating patients about oral health problems and the importance of oral health can significantly improve oral hygiene behaviours of older patients. Similarly, the common preventive measure such as application of fluoride may decrease the incidence of caries also among older people. However, there is currently a gap in information and research around effective oral health care treatments and programs in geriatric dental care and how this can be integrated into overall health management. Due to the lack of policies and guidelines, there is uncertainty regarding how oral health care can be integrated into geriatric medical care. Therefore, further research is warranted to assess the effectiveness of interventions in improving the oral health status in the elderly, and based on such evidence, efforts must be invested in developing guidelines to assist both dental and medical health care professionals in integrating good oral health as part of healthy ageing. There is no overall health without oral health.

## Data Availability

The data supporting the conclusion of this article is included within the article.
